# Optimal dose reduction algorithm using an attenuation-based tube current modulation method for cone-beam CT imaging

**DOI:** 10.1371/journal.pone.0192933

**Published:** 2018-02-15

**Authors:** Kihong Son, Jieun Chang, Hoyeon Lee, Changhwan Kim, Taewon Lee, Seungryong Cho, Sohyun Park, Jin Sung Kim

**Affiliations:** 1 Department of Nuclear and Quantum Engineering, Korea Advanced Institute of Science and Technology, Daejeon, Korea; 2 Department of Radiation Oncology, Yonsei Cancer Center, Yonsei University College of Medicine, Yonsei University Health System, Seoul, Korea; Stanford University, UNITED STATES

## Abstract

To reduce the radiation dose given to patients, a tube current modulation (TCM) method has been widely used in diagnostic CT systems. However, the TCM method has not yet been applied to a kV-CBCT system on a LINAC machine. The purpose of this study is to investigate if a TCM method would be desirable in a kV-CBCT system for image-guided radiation therapy (IGRT) or not. We have developed an attenuation–based TCM method using prior knowledge from planning CT images of patients. The TCM method can provide optimized dose reductions without degrading image quality for kV-CBCT imaging. Here, we investigate whether or not our suggested TCM method is desirable to use in kV-CBCT systems to confirm and revise the exact position of a patient for IGRT. Patients go through diagnostic CT scans for RT planning; therefore, using information from prior CT images can enable estimations of the total X-ray attenuation through a patient’s body in a CBCT setting for radiation treatment. Having this planning CT image allows to use the proposed TCM method in RT. The proposed TCM method provides a minimal amount of current for each projection, as well as total current, required to reconstruct the current modulated CBCT image with an image quality similar to that of CBCT. After applying a calculated TCM current for each projection, projection images were acquired and the current modulated CBCT image was reconstructed using a FDK algorithm. To validate the proposed approach, we used a numerical XCAT phantom and a real ATOM phantom and evaluated the performance of the proposed method via visual and quantitative image quality metrics. The organ dose due to imaging radiation was calculated in both cases and compared using the GATE simulation toolkit. As shown in the quantitative evaluation, normalized noise and SSIM values of the TCM were similar to those of conventional CBCT images. In addition, the proposed TCM method yielded comparable image quality to that of conventional CBCT images for both simulations and experimental studies as organ doses were decreased. We have successfully demonstrated the feasibility and dosimetric merit of a prototypical TCM method for kV-CBCT via simulations and experimental study. The results indicate that the proposed TCM method and overall framework can be a viable option for CBCT imaging that utilizes an optimal dose reduction without degrading image quality. Thus, this method reduces the probability for side effects due to radiation exposure.

## Introduction

Image-guided radiation therapy (IGRT) has been extensively used for target localization, patient positioning, and external beam adjustment in radiation therapy procedures [1]. One approach of IGRT is kV cone-beam computed tomography (CBCT), which is used to get a patient’s anatomy before treatment [1, 2]. In practice, kV-CBCT imaging enables technicians and doctors to observe the target and position of organs at risk before treatment while the patient is on the couch. However, repeated use of kV-CBCT has raised concerns regarding radiosensitive organs [3, 4]. A high radiation dose increases the risk of side effects in organs at risk [1–3]. Thus, there is a limit to the number of allowable CBCT images due to the patient’s radiation burden during treatment.

Accordingly, dose reduction is important and has been a major concern in reducing the risk of radiation poisoning. Various methods have been proposed to reduce the radiation dose from CBCT. These include decreasing the projection range using a collimator, rotating the x-ray source in different direction to avoid covering the critical organ, and reducing the scanning length, among others. However, these methods do not consider the size and shape of the patient.

To reduce the radiation dose given to patients, a tube current modulation (TCM) method has been widely used in diagnostic CT systems. This technique involves an adaptive tube current that is dependent on the integrated attenuation coefficient over a projection angle. This reduces the imaging dose while preserving optimal image quality [5–10]. Gies et al. [11, 12]have previously suggested an approach to apply TCM in CT by attenuating a body through a central axis to homogenize the noise by modulating X-ray intensities. Parsons et al. [13]have applied this TCM technique to a CBCT imaging system that included a four-blade dynamic kV collimator. This application was capable of reducing dose and image noise while providing high SNR values compared to those of conventional CBCT by adjusting fluence as a function of projection angle. However, due to limitations on the target imaging area, the technique could not easily confirm and revise the patient’s position. In addition, information of the desired doses for particular image quality is not accessible.

A kV-CBCT acquires a volumetric image for a single rotation in order to confirm and revise the patient’s position. Therefore, the TCM method has not yet been applied to a kV-CBCT system on a LINAC machine in a clinic. The purpose of this study is to investigate if a TCM method is desirable in kV-CBCT systems to confirm and revise the exact position of a patient for IGRT or not. We propose a framework for applying the developed TCM method to kV-CBCT systems.

To do so, we have developed an optimized attenuation–based TCM method using prior information from treatment planning CT images. This method provides an optimal radiation dose reduction without degrading image quality for kV-CBCT imaging. The proposed TCM method has been validated using both a numerical XCAT phantom and ATOM phantom. This framework has the potential to enable application of a TCM method in CBCT systems to reconstruct CT images of similar image quality to that of conventional methods while minimizing the total radiation dose. Thus, our study is expected to reduce the side effects of radiation exposure for patients in need of X-ray imaging.

## Materials and methods

### A. TCM framework

Patients were put through a diagnostic CT scan for RT planning. Using this information, one can estimate the total attenuation of an X-ray through a patient’s body in a CBCT setting for radiation therapy. To apply the proposed TCM method to CBCT scans, previously scanned CT images for treatment planning were used as follows:

1. One slice of a treatment planning CT image that included the region of interest was chosen for processing.
2. Using Siddon’s method [14], the amount of attenuation (*A*_*i*_) for each projection was calculated in the chosen slice of a CT image.
3. The proposed TCM method calculated a minimum amount of current for each projection,
4. By applying the calculated TCM current for each projection, projection images were acquired and the CT image was reconstructed using a FDK algorithm.

We performed a numerical study incorporating major factors into account such as polychromatic X-rays, scatters, noise, and bow-tie filters in order to demonstrate that the TCM method can produce images of equivalent quality to that of CBCT using reduced imaging radiation doses [15]. The CT projector is a program provided with the XCAT phantom, which takes the numerical phantom as an input and generates projection data by angle. Using the CT projector program, 680 projection images of a XCAT phantom were obtained via a conventional scanning condition—i.e., without modulating the tube current—and the proposed TCM scanning condition for a simulation study. Alternatively, a patient’s actual CT image of an ATOM phantom was used for an experimental study.

To generate a matrix of linear attenuation coeffients, we analyzed a 60 keV photon from the NIST because it was approximately within a mean energy of a 120 kVp spectrum of a X-ray tube on pelvis mode from a XVI-CBCT. Projection images were generated in 1^◦^ increments to achieve a full rotation around the phantom for both conventional CBCT and TCM scans.

### B. Calculating the current of TCM to minimize radiation dose

Gies et al. studied the theoretical prediction of the TCM method, revealing that dose control via TCM has the potential to either improve image quality through noise reduction or reduce radiation exposure without impairing image quality [11]. Therefore, we developed a formulation based on that studied by Gies et al. to determine variations in fluence as a function of projection angle. Briefly, this calculation is described using the following equation from Gies et al.:

$$N_{0i}=\frac{N_{0}}{\sum_{i=1}^{P}\sqrt{A_{i}}}\cdot \sqrt{A_{i}}$$ (1)

where *N*_*oi*_ is the required number of quanta after passing through the phantom at an angle *i*, *No* is the total number of emitted quanta throughout an acquisition (total current), *P* is the number of projections, and *A*_*i*_ is the amount of attenuation through the phantom at angle *i*. *N*_*i*_ is the number of quanta in view *i*, after traversing the object. Thus, the attenuation of the in-view, *i*, is given by *A*_*i*_ = $\frac{N_{0i}}{N_{i}}$.

Then, the pixel noise variance of the image without a TCM case is calculated using

$$\sigma^{2}=\sum_{i=1}^{P}\sigma_{i}^{2}=\sum_{i=1}^{P}\frac{A_{i}}{N_{0i}}$$ (2)

Substituting Eq (1) for Eq (2), the level of noise variance for a TCM-generated image is calculated using

$$\sigma^{2}=\frac{(\sum_{i=1}^{P}\sqrt{A_{i}}{)}^{2}}{N_{0}}$$ (3)

From the above equations, we have devised a method that minimizes the total current for any given image quality using the TCM technique. To preserve an equivalent image quality to that of conventional CBCT, setting Eq (2) equal to Eq (3) yields Eq (4), as follows:

$$\sigma_{CBCT}^{2}=\sum_{i=1}^{P}\frac{A_{i}}{N_{0i}}=\frac{{\left(\sum_{i=1}^{P}\sqrt{A_{i}}\right)}^{2}}{N_{0}^{\prime }}=\sigma_{TCM}^{2}$$ (4)

From this equation, the total current, $N_{o}^{\prime }$, could be generated to preserve image quality while minimizing dose, as follows:

$$N_{0}^{\prime }=\frac{(\sum_{i=1}^{P}\sqrt{A_{i}}{)}^{2}}{\sum_{i=1}^{P}\frac{A_{i}}{N_{0i}}}$$ (5)

Finally, substituting Eq (5) for Eq (1), the minimized tube current, $N_{0}^{\prime }$, for each scanning angle can be written as Eq (6).

$$N_{0i}^{\prime }=\frac{N_{0}^{\prime }}{\sum_{i=1}^{P}\sqrt{A_{i}}}\;\sqrt{A_{i}}$$ (6)

**Fig 1:**
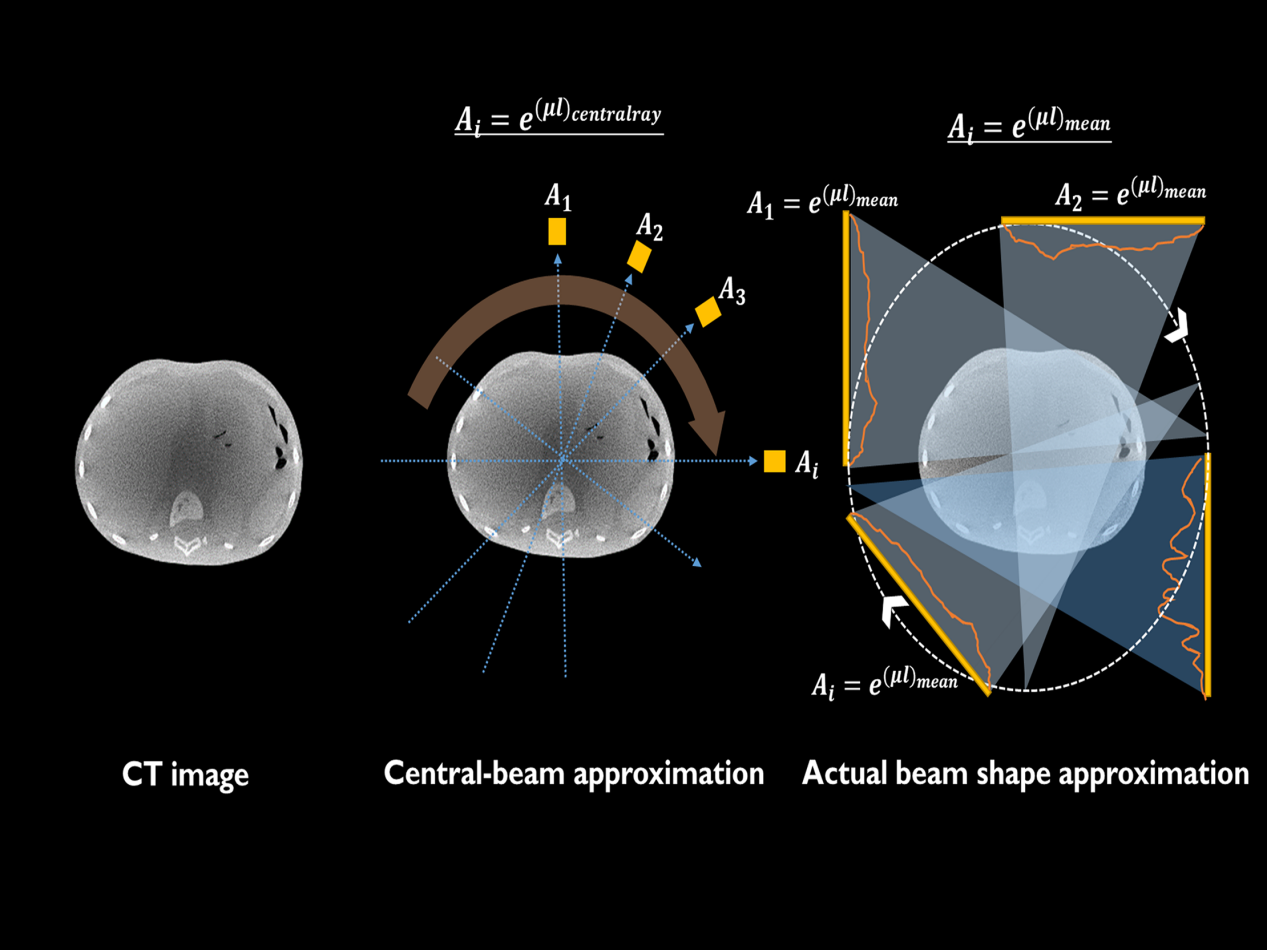
Framework for calculating Ai values. After selecting the CT image as in (a), we calculated the Ai value by two methods (b) Central-beam approximation and (c) actual beam shape approximation.

Therefore, from the above equation, the method of minimizing the total current is finally derived to make a given image quality using the TCM technique. As Fig 1 shows, the amount of attenuation (*A*_*i*_) was calculated in two ways: the central beam line attenuation was calculated and compared to the average attenuation over certain lines that were chosen by considering the beam shape on the detector.

### C. Simulation study

#### C.1 OBI kV-CBCT system

The kV X-ray beams can be delivered in both half-fan and full-fan modes of a Varian on-board imager (OBI) system mounted on a linear accelerator [1, 16]. The full-fan mode is a default mode for the head-and-neck region because of its narrow field-of-view (FOV); whereas, the half-fan mode, due to its increased effective FOV size, is used for most other regions such as the chest, abdomen, and pelvis regions. For the simulation study, we used a pelvis mode with a half-fan mode. This scanning mode uses a half-fan bow-tie filter and a scanning angle of 360°.

**Fig 2:**
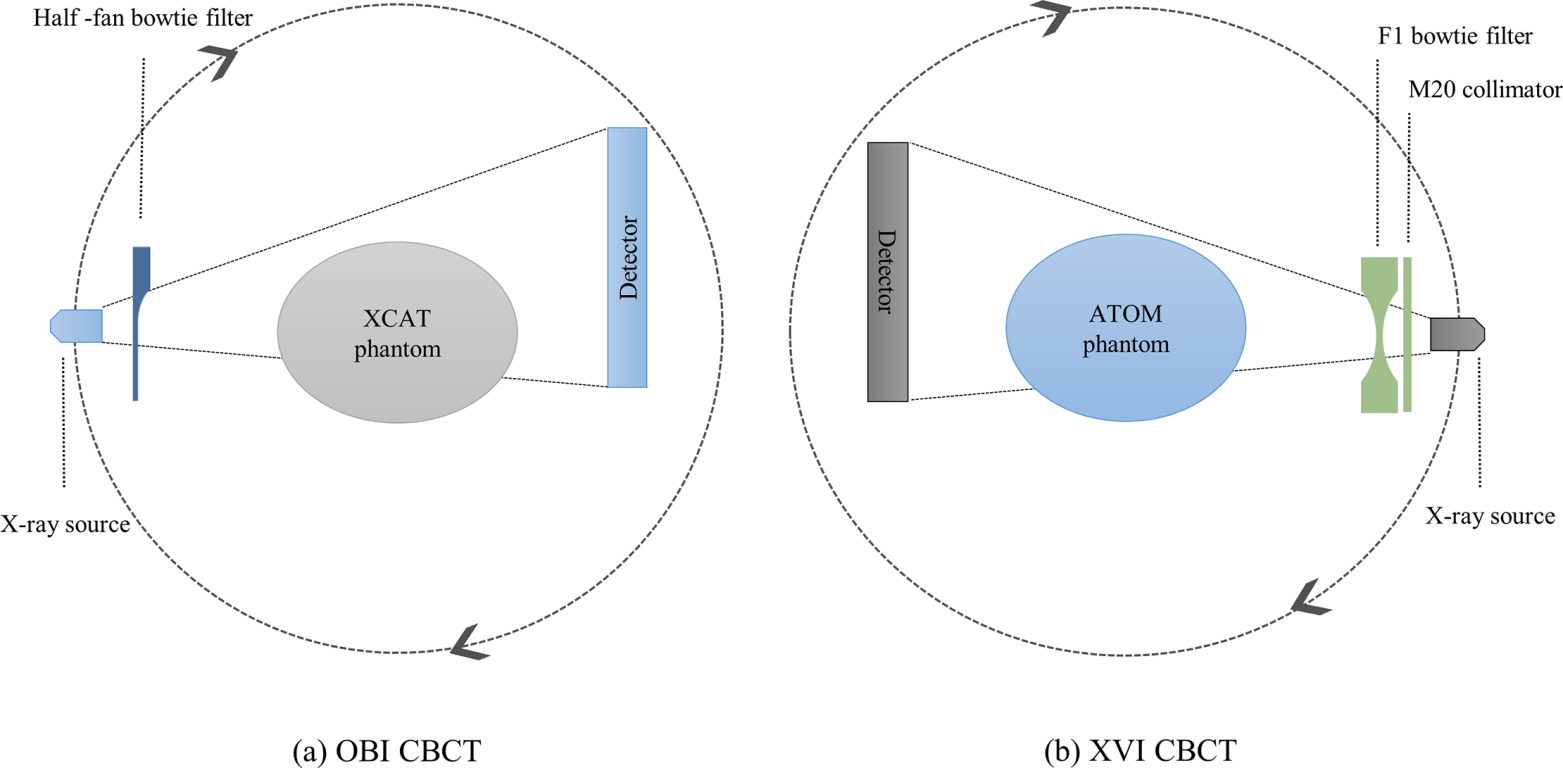
Half-fan scanning geometry. (a) OBI CBCT and (b) XVI CBCT system. The default detector position shifted 14.8 cm using the OBI and 11.5 cm for the XVI in medium-FOV mode.

The pelvis mode was operated at its own default exposure condition, with a voltage of 125 kVp and a total current of 681 mAs. A schematic of the pelvis mode is shown in Fig 2. We have estimated the dose distribution and organ doses using a MC simulation for a conventional CBCT scan and TCM case, respectively.

The XCAT phantom, which is a realistic and flexible anatomical model of the whole human body for the study of medical imaging and an important tool for evaluating imaging devices and techniques. In this study, the XCAT phantom was incorporated to calculate the absorbed radiation doses in organs at risk and validate the proposed TCM method for CBCT [17–20].

#### C.2 Monte carlo simulation

The organ dose due to imaging radiation has been calculated using the GATE v.6 simulation toolkit [21].

A Varian OBI kV-CBCT system was implemented according to system specifications. In addition, various radiation physics were included in this simulation such as the photoelectric effect, Compton scattering, Rayleigh scattering, electron ionization, Bremsstrahlung, and multiple scattering of electrons. The beam quality and quantity have been confirmed by comparing the weighted computed tomography dose index (CTDI_w_) values reported outlined by the Varian Medical Systems [22, 23].

### D. Experimental study

#### D.1 XVI kV-CBCT system

Our Elektra System has a XVI CBCT system (Elekta, Crawley, UK) [2, 4]. There were two filter cassettes available for the unit: the F0 neutral filter and F1 bowtie filter. All cassettes had thin plastic windows at the top and bottom. The F1 bowtie filter was made of an aluminum alloy, and the shape of the bowtie was doubly concave in the cross-line direction and flat about the central beam axis in the inline direction.

There were also three types of collimator cassettes used: Small, Medium, and Large. Each type produced a small, medium, or large field of view (FOV), respectively, wherein the different FOVs were defined by the panel offset from the central axis of the kV. The F1 and M20 filter cassette and collimator type, respectively, were used in this study. The pelvis scan mode in the XVI—whose default projection conditions were given as 120kVp, 3.125 mAs—was adopted for 360 projection views per angle in this study.

#### D.2 The ATOM phantom

**Fig 3:**
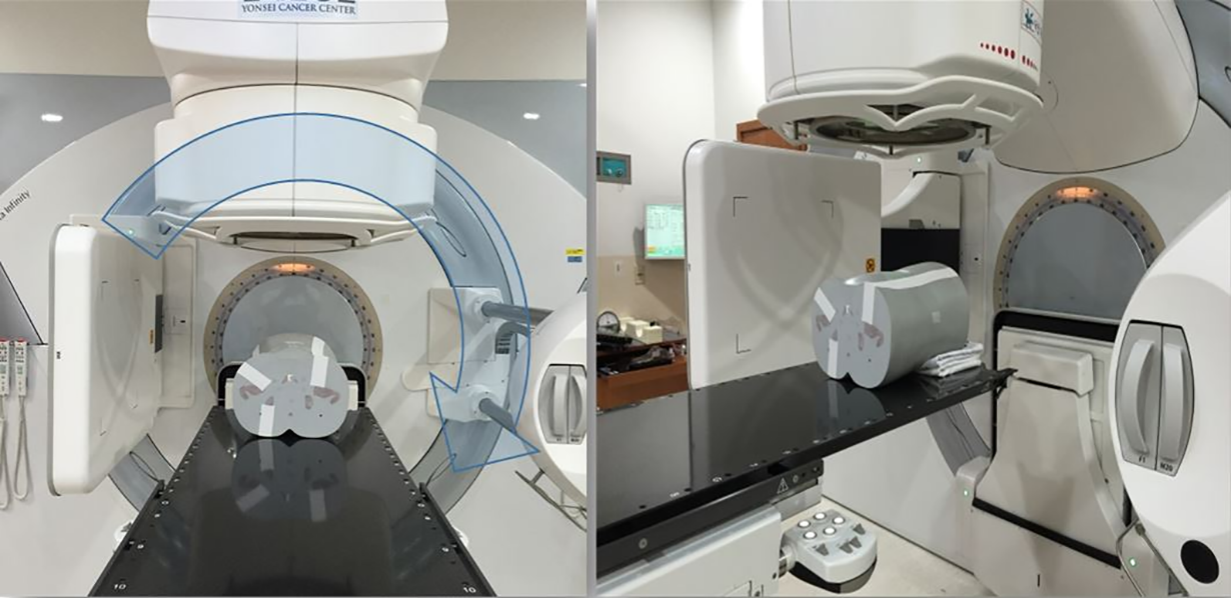
The Elekta XVI-CBCT system and CIRS ATOM phantom.

CIRS ATOM phantoms are a full line of anthropomorphic, cross-sectional dosimetry phantoms designed to investigate organ dose, whole body effective dose, and evaluate image quality [24]. We selected the adult male phantom for this study. This phantom is sectional in design, with traditional 25 mm thick sections. Fig 3 shows the XVI CBCT and ATOM phantom.

## Results

### A. Simulation results

**Fig 4:**
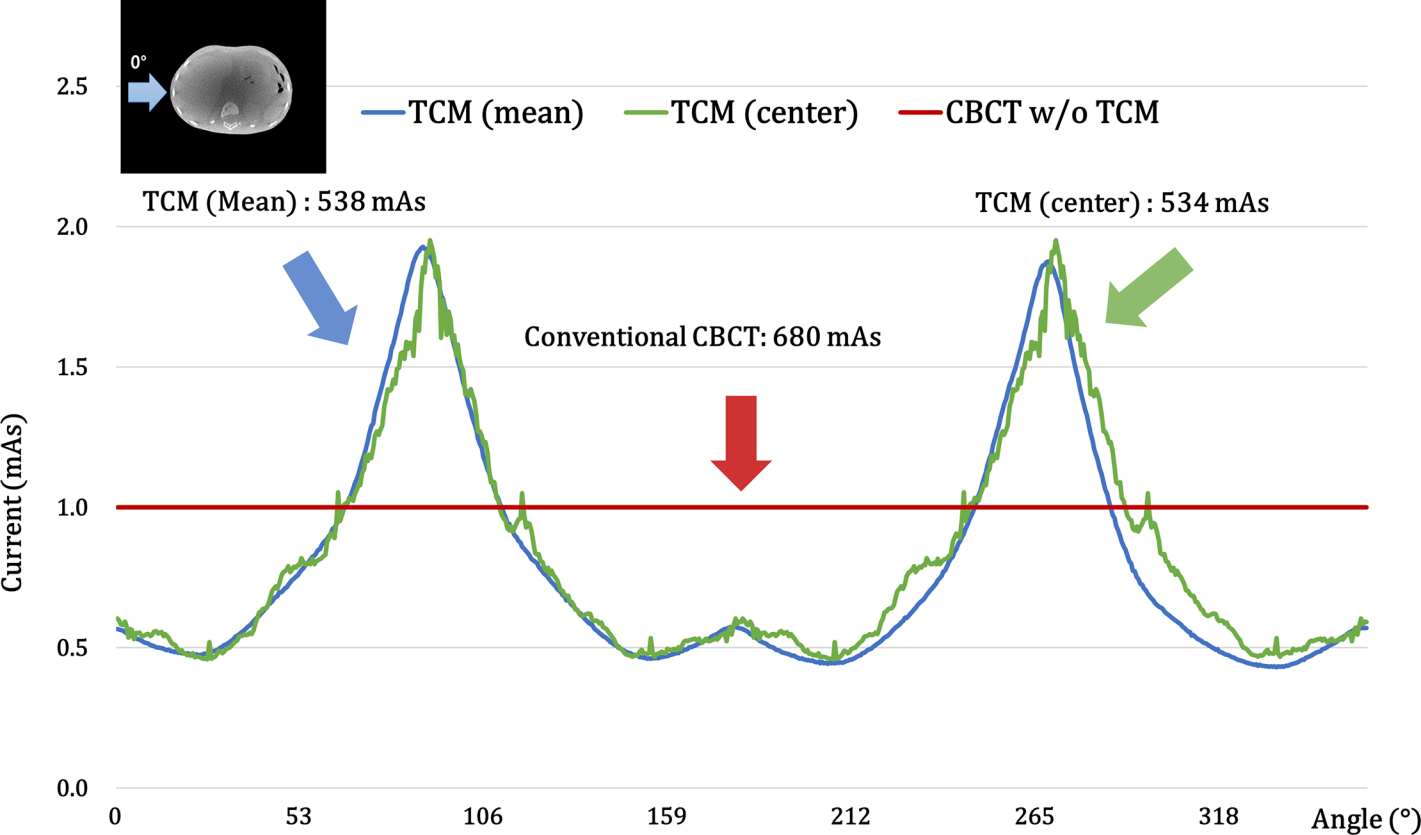
TCM dose per angle for simulation study. The red line shows a conventional CBCT scan, the green-line represents a TCM scan from a center-beam approximation, and the blue-line indicates a TCM scan of an actual beam shape approximation.

In Fig 4, the optimum TCM current for each projection angle was plotted for the XCAT’s abdominal region. The current was modulated for each projection angle to provide more current for higher attenuation angles and less current for lower attenuation angles.

**Fig 5:**
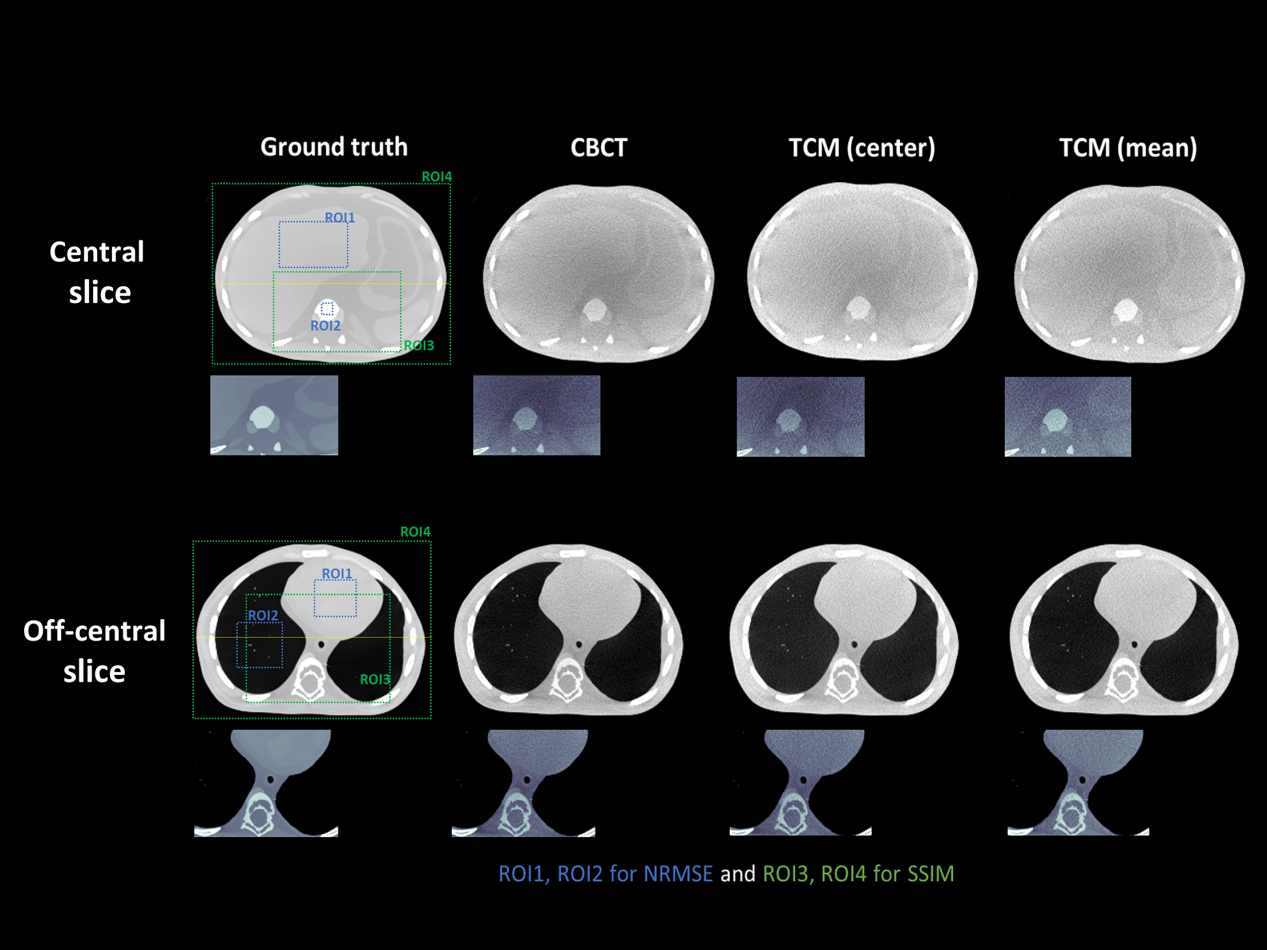
Reconstructed images of the XCAT phantom for central and off-center slices from conventional CBCT and TCM methods.

Fig 5 shows a conventional CBCT image and TCM image for both central and off-center slices. Although the total amount of current for the TCM image was reduced by 21% compared to the conventional image, there was little difference between the reconstructed images. The normalized root-mean square error (NRMSE) and structure similarity (SSIM) index were calculated to investigate the quantitative accuracy of the images. The NRMSE used in this study was defined by

$$\mathrm{NRMSE}=\mu^{-1}\sqrt{\frac{1}{N}\sum_{i=1}^{N}{\left(x_{i}-\mu \right)}^{2}}$$ (7)

where *μ is* mean value of the ROI; *x_i_* signified the pixel values in the ROI; and *N* is the number of pixels within the ROI. A structure similarity index was used to measure the degree of similarity between the reference image and an image of interest. The SSIM is sensitive to contrast, luminance, and structures within an image. Its value ranges from 0 to 1. As the SSIM value gets closer to 1, the similarity between the reference and image of interest increases [25]. Table 1 shows reconstructed CT images using the TCM method had comparable NRMSE properties and similar SSIM values to the reference images acquired via conventional CBCT at both central and off-center slices. In addition, the reconstructed TCM images exhibited small difference between the central beam approximation and actual beam shape approximation. Reconstructed images from the actual beam shape approximation had similar values to those from a conventional method.

**Table 1:** NRMSE and SSIM values are calculated for center and off-center slices. ROIs are defined in Fig 5.

**Center slice**	**NRMSE**		**SSIM**	
	ROI1 (tissue)	ROI2 (bone)	ROI3	ROI4 (all)
**Ground**	0.0112	0.0037	-	-
**CBCT**	0.0686	0.0396	0.9875	0.9997
**TCM_center**	0.0740	0.0427	0.9866	0.9996
**TCM_mean**	0.0691	0.0404	0.9943	0.9999
**Off-center slice**	**NRMSE**		**SSIM**	
	ROI1 (heart)	ROI2 (lung)	ROI3	ROI4 (all)
**Ground**	0.0240	0.0680	-	-
**CBCT**	0.0449	0.0978	0.9989	0.9995
**TCM_center**	0.0483	0.1051	0.9989	0.9995
**TCM_mean**	0.0449	0.1047	0.9995	0.9997

### B. Calculating organ dose

**Fig 6:**
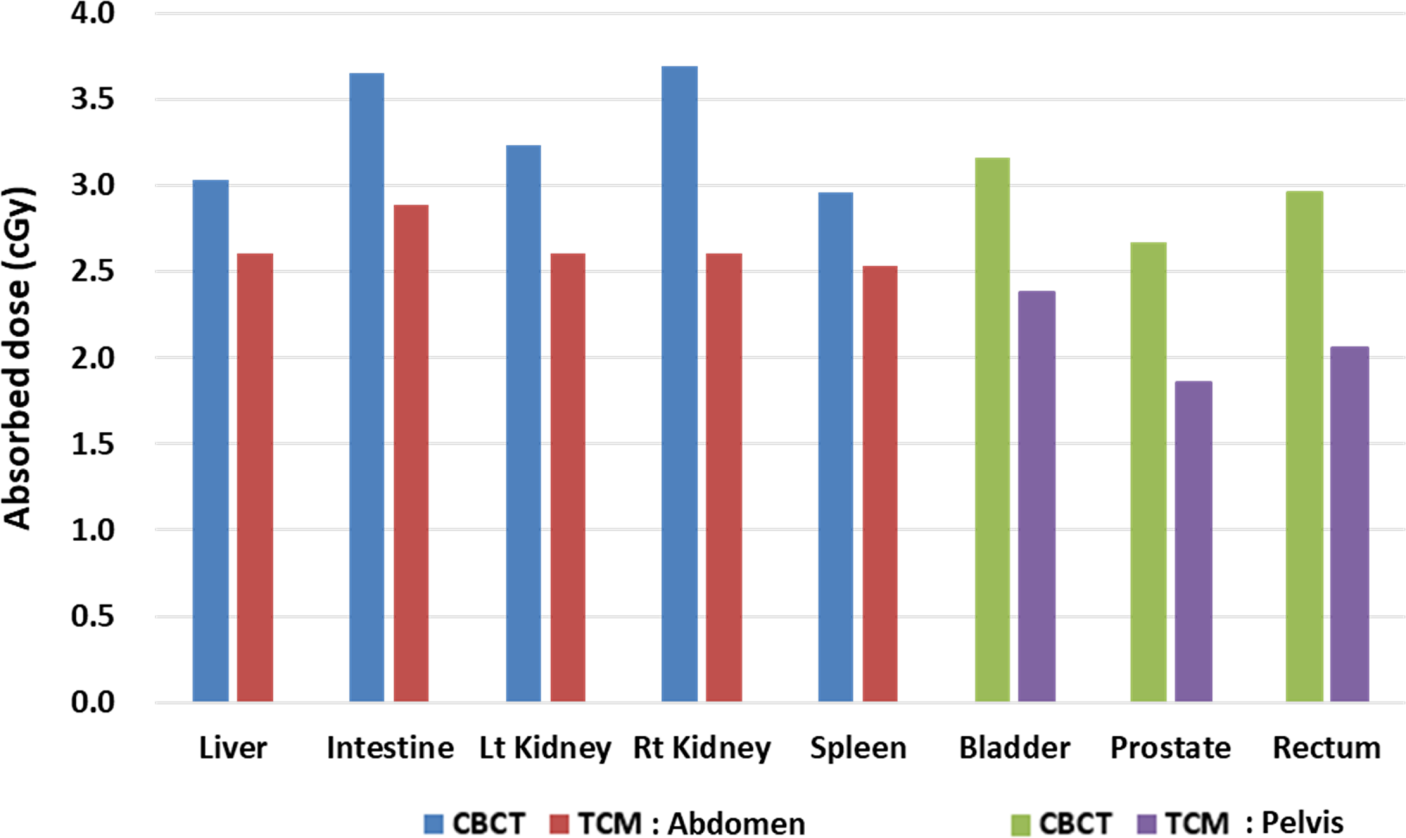
Imaging dose to abdominal and pelvic organs from conventional CBCT and TCM scans using a XCAT phantom.

The imaging doses for abdominal and pelvic organs are shown in Fig 6. Using the TCM scans in the pelvis mode, a reduction in organ doses ranged from 14% to 31% in comparison to conventional CBCT scans. The organ doses were calculated to range from 2.67 to 3.69 cGy for the conventional CBCT scan, and 1.95 to 2.97 cGy for the TCM scan. Those from the TCM scans were around 20% lower for abdominal organs and 29% lower for pelvic organs when compared to the conventional CBCT mode.

### C. Experimental results

**Fig 7:**
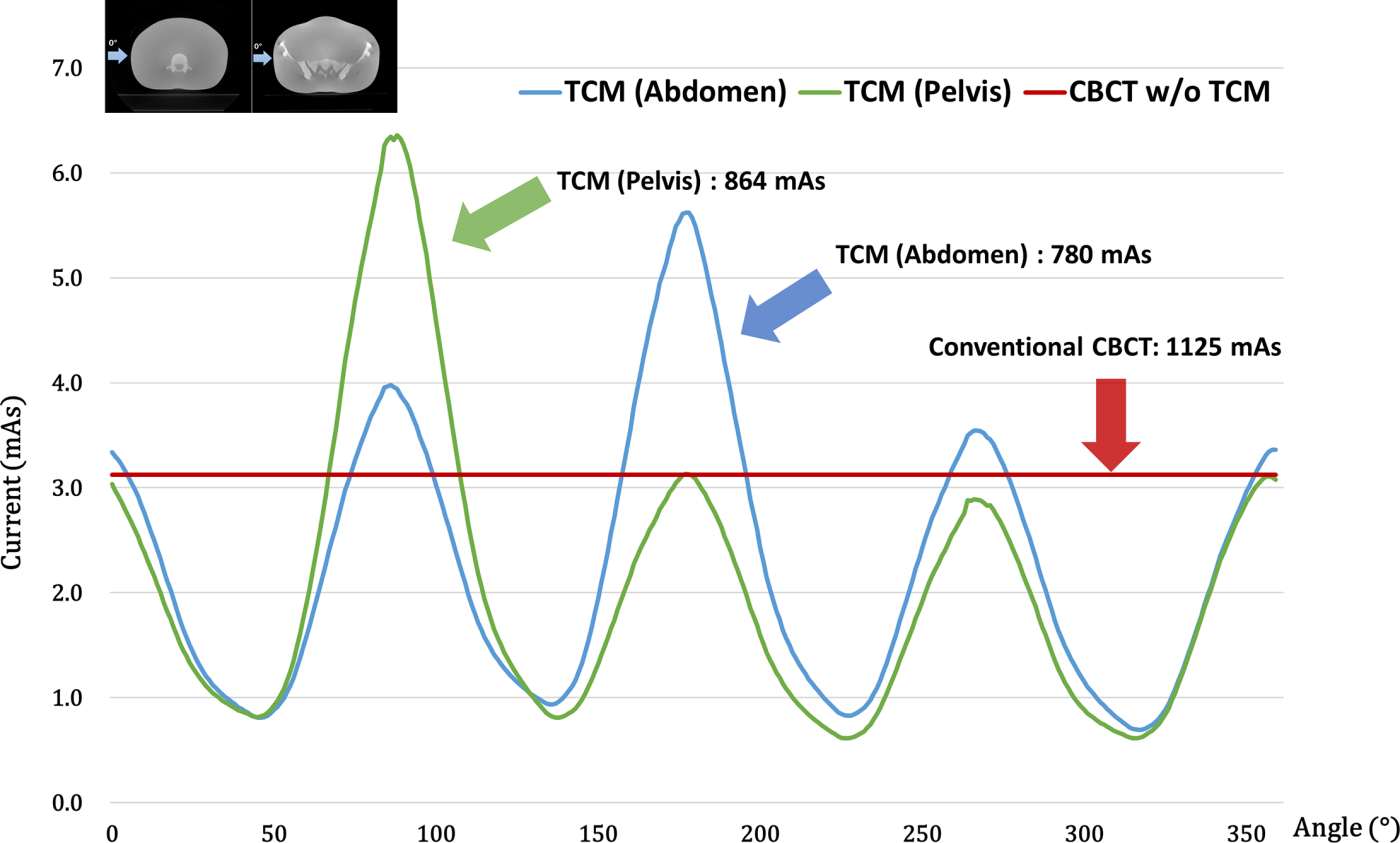
TCM dose per angle is calculated for the abdominal and pelvic regions of the ATOM phantom. The red line shows the results of a conventional CBCT scan; the green line represents the TCM scan of the pelvic region; and the blue line signifies the TCM scan of the abdominal region.

In Fig 7, we calculated the optimal TCM current and projection angle for the ATOM abdominal and pelvic regions shown in Fig 1. We conducted an experimental study using TCM to get an actual beam shape approximation. As shown in the simulation study, variations in the amount of current along with projection angle were observed. Compared with a symmetrically current-modulated abdominal region, we found asymmetry in the pelvic region due to the greater number of bones in this region.

**Fig 8:**
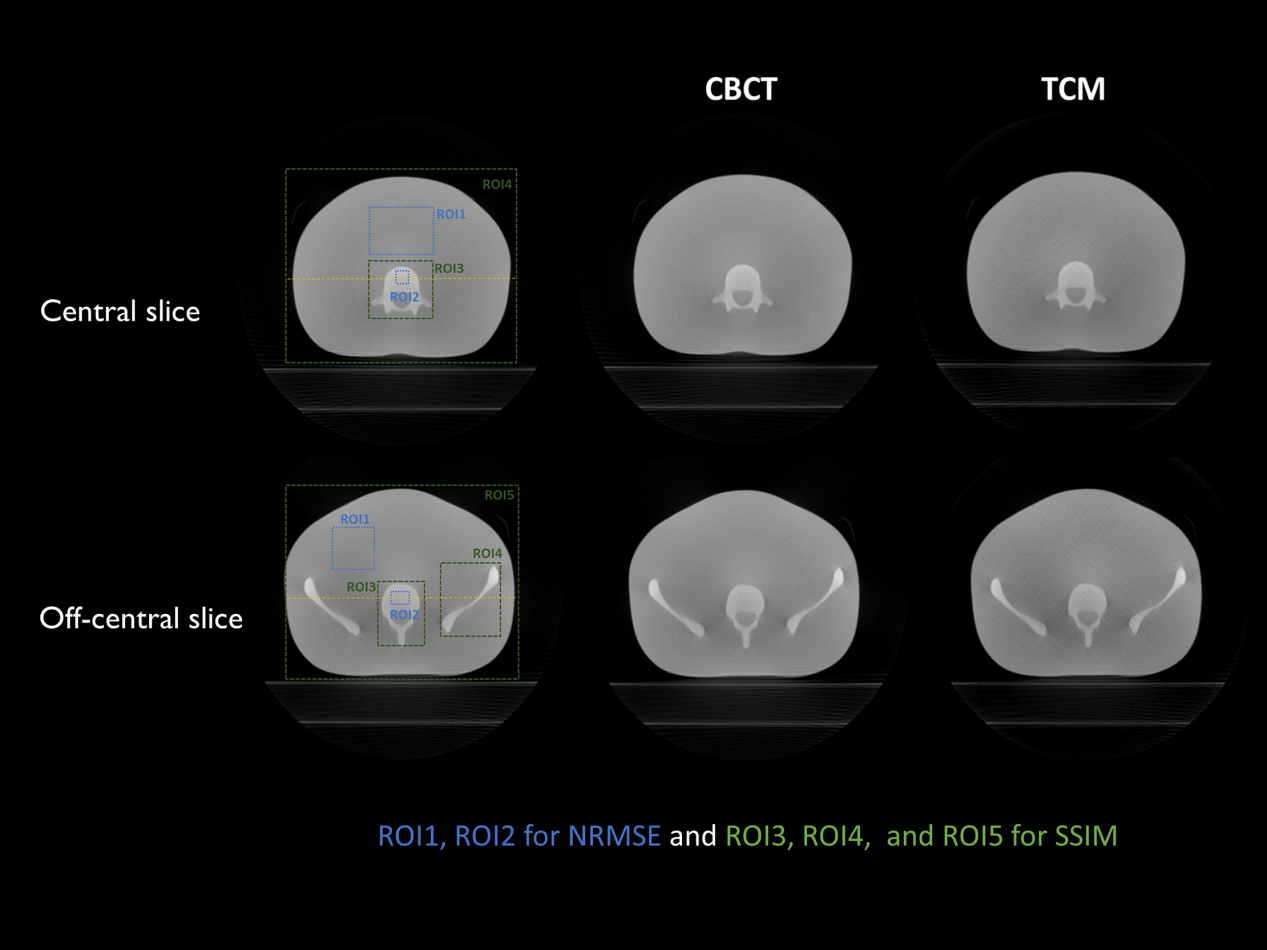
Reconstructed images of the ATOM’s abdominal region using conventional CBCT and TCM methods. XVI images are obtained via central and off-center slices.

**Fig 9:**
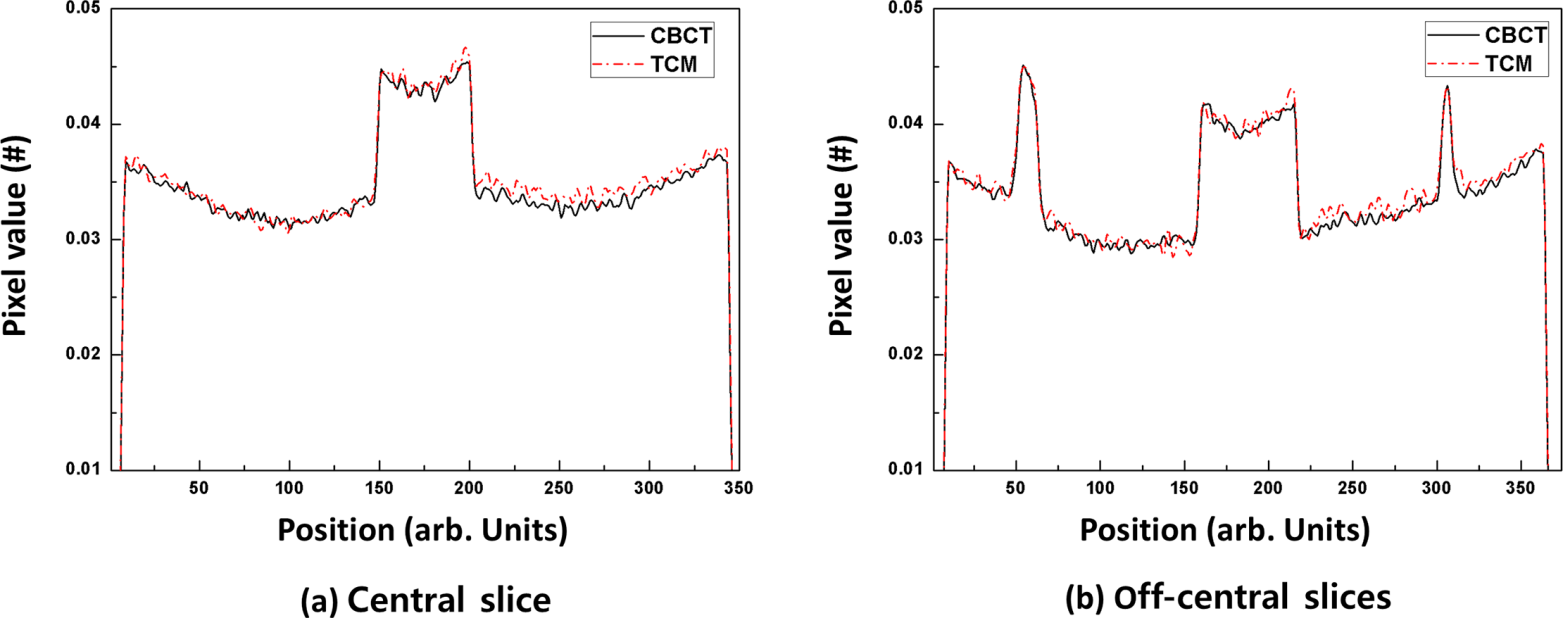
Line profiles from Fig 8. A conventional CBCT image is plotted as a solid black line; the TCM images are plotted as dash-dot red lines.

Fig 8 shows a conventional CBCT image and TCM image of an ATOM abdominal region for central and off-center slices. Although the total dose for the TCM image was reduced by 23% compared with that of the conventional image, both images exhibited comparable image quality for image guidance. The line profiles for the dashed lines in Fig 8 are shown in Fig 9. The line profile of the reconstructed image using the TCM method is plotted as a dash-dot red line. The TCM image had a similar value to that of the conventional CBCT image.

**Fig 10:**
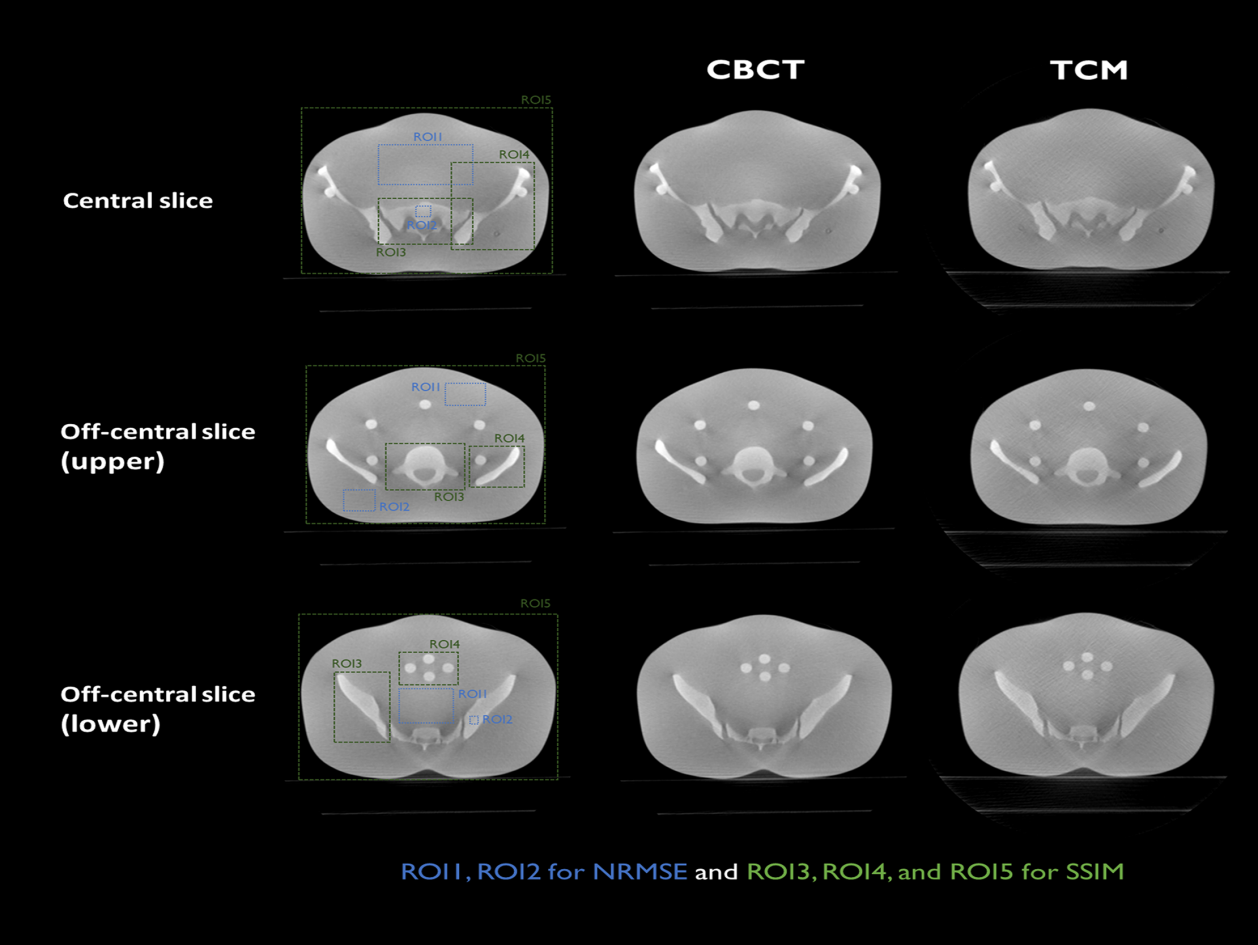
Reconstructed images of ATOM’s pelvic region from conventional CBCT and TCM methods. XVI images are from the central, upper, and lower off-center regions.

As shown in Table 2, there is little difference in terms of NRMSE values for soft tissue and bone between the TCM and conventional images. SSIM values of the selected ROIs were very close to 1. Moreover, the reconstructed images of the ATOM pelvic region from conventional CBCT and the TCM method are shown in Fig 10. Here, the three parts comprise the central slice, off-center slice of the upper region, and off-center slice of the lower region. Even though the total dose for the TCM image was reduced by 31%, the image itself was a little noisier than the CBCT image, but the detailed structures were similar. The quantitative evaluation in Table 3 shows that NRMSE values of the TCM exhibited little difference when compared with those of the CBCT. SSIM values confirmed that the TCM image was comparable to the conventional CBCT image.

**Table 2:** NRMSE and SSIM are calculated for center and off-center slices. ROIs are defined in Fig 8.

ATOM Abdomen		Center slice		Off-center slice		
		ROI1 (tissue)	ROI2 (bone)	ROI1 (tissue)	ROI2 (bone)	
**NRMSE**	**CBCT**	0.0192	0.0315	0.0212	0.0247	
	**TCM**	0.0207	0.0329	0.0219	0.0266	
		ROI3 (bone)	ROI4 (all)	ROI3 (bone1)	ROI4 (bone2)	ROI5 (all)
**SSIM**	**TCM**	0.9996	0.9999	0.9998	0.9999	0.9999

**Table 3:** NRMSE and SSIM calculated for central and off-center slices. ROIs are defined in Fig 10.

ATOM Abdomen		Center slice			Off-center slice (upper)			Off-center slice (lower)		
		ROI1 (tissue)	ROI2 (bone)		ROI1 (center)	ROI2 (side)		ROI1 (tissue)	ROI2 (bone)	
**NRMSE**	**CBCT**	0.0308	0.0304		0.0161	0.0182		0.0495	0.0160	
	**TCM**	0.0309	0.0302		0.0171	0.0185		0.0505	0.0155	
		ROI3 (bone1)	ROI4 (bone2)	ROI5 (all)	ROI3 (bone1)	ROI4 (bone2)	ROI5 (all)	ROI3 (bone1)	ROI4 (bone2)	ROI5 (all)
**SSIM**	**TCM**	0.9977	0.9984	0.9999	0.9984	0.9971	0.9997	0.9962	0.9952	0.9997

## Discussion

Gies et al. [11] have previously described a method to calculate TCM in CT based on the attenuation through a central axis, resulting in a desired number of quanta at the detector plane for every projection. This shows that both dose and image noise could be reduced. To the best of our knowledge, there is no precedent where this TCM method is applied to a CBCT system for clinical applications.

Parsons et al. [13] recently showed that the TCM method could be successfully applied to kV-CBCT using a collimator to image the target region. Doing so demonstrated a reduction in noise values compared to those from conventional kV-CBCT. However, due to a limited target area, there were limitations in the ability to confirm and revise the patient’s position. Moreover, proper dose information to yield high image quality has not been accessible.

In our study, we calculated the amount of attenuation for each projection angle, taking into account the actual beam shape of the CBCT, based on the patient’s CT image. We then reconstructed the image for a desired image quality, without truncation. To do so, we calculated the minimum amount of TCM current needed to reconstruct an image with our desired quality. The results indicate that the proposed TCM method yielded comparable image quality to that from conventional CBCT images, while organ doses were significantly lower using TCM. Thus, radiation risk could be decreased by minimizing the required dose needed to reconstruct a CBCT image.

The potential to apply TCM in a CBCT system was demonstrated with our proposed framework. We used a previously acquired plan CT images for the TCM method so that additional radiation exposure could be avoided. We also calculated patient-specific TCM currents based on attenuation. We reconstructed the entire area of each image rather than a partially targeted area, thereby increasing clinical efficiency.

Our study can be applied not only to kV-CBCT on LINAC for radiation therapy, but also to C-arm CT for interventional radiology. However, the proposed method requires a CT image taken in advance to obtain the tube current by angle. Maybe it is possible to modulate the beam current based on the patient’s size and shape which wouldn’t require an input CT image.

We chose the central slice as our region of interest and calculated the TCM dose based on a central beam approximation or actual beam-shape approximation. In the future, we will calculate the TCM dose based on a CBCT 3D volumetric beam shape to improve the accuracy of the TCM image. Furthermore, we believe that combining this technique with noise reduction or iterative reconstruction algorithms would yield great improvements to the TCM image [2, 26–28].

## Conclusions

We have successfully demonstrated the feasibility and dosimetric merit of a TCM method for kV-CBCT via simulation and experimental study. The results substantiate the idea that the proposed TCM method and framework can be a useful option for CBCT imaging that provides optimized dose reductions without degrading image quality. Therefore, the probability for side effects due to radiation exposure can be significantly decreased.
